# Time-Resolving Study of Stress-Induced Transformations of Isotactic Polypropylene through Wide Angle X-ray Scattering Measurements

**DOI:** 10.3390/polym10020162

**Published:** 2018-02-08

**Authors:** Finizia Auriemma, Claudio De Rosa, Rocco Di Girolamo, Anna Malafronte, Miriam Scoti, Geoffrey Robert Mitchell, Simona Esposito

**Affiliations:** 1 Dipartimento di Scienze Chimiche, Università di Napoli “Federico II”, Complesso Monte Sant’ Angelo, via Cintia, 80126 Napoli, Italy; claudio.derosa@unina.it (C.D.R.); rocco.digirolamo@unina.it (R.D.G.); anna.malafronte@unina.it (A.M.); miriam.scoti@unina.it (M.S.); Simona.Esposito@lamberti.com (S.E.); 2 CDRSP—Centre for Rapid and Sustainable Product Development, Polytechnic Institute of Leiria, Centro Empresarial da Marinha Grande, 2430-028 Marinha Grande, Portugal; geoffrey.mitchell@ipleiria.pt

**Keywords:** isotactic polypropylene, stress-induced phase transitions, structural analysis, X-ray diffraction

## Abstract

The development of a highly oriented fiber morphology by effect of tensile deformation of stereodefective isotactic polypropylene (iPP) samples, starting from the unoriented γ form, is studied by following the transformation in real time during stretching through wide angle X-ray scattering (WAXS) measurements. In the stretching process, after yielding, the initial γ form transforms into the mesomorphic form of iPP through mechanical melting and re-crystallization. The analysis of the scattering invariant measured in the WAXS region highlights that the size of the mesomorphic domains included in the well oriented fiber morphology obtained at high deformations increases through a process which involves the coalescence of the small fragments formed by effect of tensile stress during lamellar destruction with the domain of higher dimensions.

## 1. Introduction

The structural and morphological transformations of isotactic polypropylene (iPP) by effect of stretching have been widely studied [1,2,3,4,5,6,7,8,9,10,11,12,13,14,15,16,17,18,19,20]. These studies are mainly concerned with iPP samples synthesized by Ziegler-Natta (ZN) heterogeneous catalyst systems [1,2,3,4,5,6,7,8,9,10,11,12,13,14,15]. In general, ZN-iPP crystallizes from the melt in the α form, which shows high rigidity, and breaks at low deformations before yielding [1,2,3,4,5,6,7,8,9,10,11,12,13,14,15,21]. Therefore, the key to probing the mechanical response of iPP, in the α form up to high deformations, generally consists in crystallizing iPP from the melt at high cooling rates, to obtain more easily deformable samples, due to formation of thin lamellar crystals or of the mesophase [1,2,3,4,5,6,7,8,9,10,14]. 

More recently, the deformation behavior of metallocene-made iPP samples with a uniform distribution of stereodefects has been also studied [16,17,18,19,20]. These samples contain only one kind of stereo-defects, namely isolated *rr* triads, at concentrations in the range 0.5–11 mol % [16]. They crystallize from the melt as mixtures of the α and γ forms [22]. In particular, the relative amount of γ form increases with increasing the concentration of *rr* defects. Simultaneously, the values of the melting temperature and the degree of crystallinity decrease from 160 to 84 °C, and from 70% to 40%, respectively, and also the values of Young modulus decrease, from 200 to 19 MPa [16,18,22]. However, in spite of the relatively high values of melting temperature, crystallinity and Young modulus, these samples show high ductility, with values of deformation at break increasing with increasing the concentration of defects [16,18,22]. They define a class of materials with properties which can be finely tuned by introducing the tailored amount of *rr* defects in the reactor step [17,22]. Typically, properties of stiff-plastic materials with high melting temperatures are achieved at low defect concentrations, whereas at intermediate *rr* concentrations, the samples show properties of flexible-materials melting in the temperature range 115–120 °C, and achieve properties of elastomeric materials with high strength due to the high crystallinity (higher than 40%), melting at 80–110 °C, at high concentration of *rr* defects [22]. This behavior is clearly different from that of ZN-iPP, for which, the γ form may be obtained only at high pressures and in low molecular mass fractions [23]. 

The study of the structural and morphological transitions occurring by effect of stretching in metallocene-made iPP samples was studied both at unit cell and lamellar length scales, by performing wide (WAXS) and small (SAXS) angle X-ray scattering measurements [16,17,18,19,20]. In particular, WAXS analysis show that during the transformation of the initially isotropic spherulitic morphology into a well oriented fiber morphology by effect of stretching, the initial crystalline α and/or γ forms transform into a well-oriented mesophase [16,17,18,19,20], as in Ziegler-Natta iPP [1,2,3,4,5,6,7,8,9,10,11,12,13,14,15]. The transformation into mesophase is gradual and starts occurring at deformations higher than the yielding point, that is concomitant with lamellar fragmentation (mechanical melting) and successive recrystallization into fibrils [17,18,19,20,24,25]. The critical strain at which the phase transition starts occurring depends on the stability of the crystal blocks initially present in the sample and the entanglement density of the amorphous phase [17,18,19,20,24,25]. SAXS analysis, instead, reveals that the main events occurring at low deformations are interlamellar separation and lamellar reorientation [19,20,26,27,28,29]. With increasing the deformation, chevron-like textures develop, followed by fragmentation of lamellae, and cavitation [19,20,29,30,31,32,33,34]. The fibrillary morphology obtained upon lamellar fragmentation is characterized by rod-like entities consisting of mesomorphic elongated aggregates separated by amorphous regions, placed at uncorrelated longitudinal and lateral distances [19]. 

In this paper, we focus on an aspect often neglected in performing the analysis of the WAXS data collected in real time during tensile deformation, that is the behavior of the scattering invariant defined in the WAXS region as a function of deformation. The analysis is illustrated in the case of two stereodefective metallocene iPP samples and has allowed highlighting a process of coalescence of mesomorphic fragments included in the well oriented fibers obtained at high deformations with consequent formation of larger domains.

## 2. Materials and Methods

Samples, synthesized using single site organometallic catalysts activated with methylalumoxane as described in ref. [35], are analyzed. The samples are characterized by molecular mass of ≈120 and ≈210 kg/mol, a polydispersity index close to two and a concentration of stereo-defects consisting solely of isolated *rr* triads equal to 5.92 and 11.01 mol % [22]. The samples show high level of crystallinity (≈40–55%), relatively high melting temperature, glass transition temperature around 0 °C, and crystallize from the melt almost completely in the γ form [22]. The main characteristics of these samples are shown in Table 1.

Specimens of rectangular shape and initial gauge length *l*_0_ of 3.0 mm, width w_0_ of 1.6 mm, and thickness *t*_0_ of 0.25 mm were cut from films obtained by melting the samples in a hot press up to reach a temperature 20–30 °C higher than the melting temperature determined in the DSC scans, using only a small pressure, and by cooling the melt to room temperature while fluxing cold water in the refrigerating system of the press (average cooling rate ≈ 10 °C/min). 

The stretching experiments are performed at 25 °C while collecting in situ bidimensional WAXS patterns using the high flux available at the Synchrotron Radiation Source in Daresbury (Cheshire, UK, beam-line 16.1). The applied deformation rate is 2.36 mm/min, the wavelength of incident radiation is λ = 1.4 Å, and the WAXS images are collected at a rate of 1 frame/5 s. The size of the primary X-ray beam at the sample position is 0.1 × 0.1 mm^2^. The strong reflections of the unoriented film specimens are used to determine the correct beam center coordinates and the sample to detector distance. Roughly, the transmission factor is determined measuring the intensity of the direct beam (using an attenuator) in absence of the sample, and in presence of the sample, before stretching, and at the end of stretching experiment. In no case the sample was brought to the rupture. Raw WAXS data are reduced and analyzed using the home made software XESA [36]. In the experiments, the X-ray beam is incident on the central region of the specimens. In this way, the structural changes occurring by effect of mere uniaxial deformation with negligible shear components may be probed. The equatorial profiles are obtained by radial integration of WAXS intensity along arcs in slices spanning an angle of ±20° with respect to the horizontal axis (equator) of the bidimensional WAXS images. We checked that in the stretching conditions adopted for the in situ WAXS measurements, the samples experience uniform deformation. We found that the thickness *t* decreases according to a power law *t* = *t*_0_ (*l*_0_/*l*)^ν^, with ν comprised between 0.4 and 0.5, depending on the deformation, *l* being the gauge length of the deformed specimens [19,20]. In the calculation we set, for the sake of simplicity, ν ≈ 0.5, in the whole deformation range, as expected for ideal rubbery materials [37].

## 3. Results

**Figure 1:**
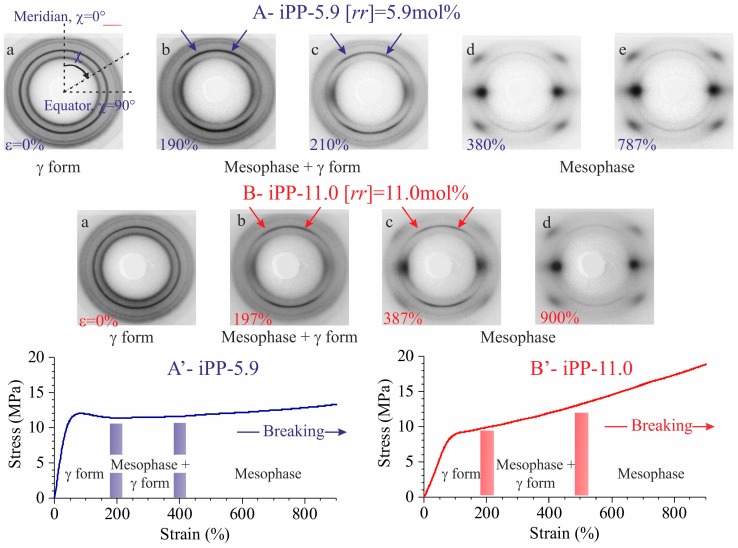
Stress strain curves (**A’**,**B’**) and bidimensional WAXS patterns of the sample iPP-5.9 (**A**) and iPP-11.0 (**B**) at the indicated deformations. The critical values of deformation marking the beginning (ε_start_), and the complete (ε_end_) transformation of the initial γ form into mesophase are indicated by the vertical bold lines in (**A’**,**B’**). The stretching direction is vertical. Arrows in b and c indicate the polarization of the (008)_γ_ reflection of γ form, off the equator (horizontal direction) and off the meridian (vertical direction), defined in the image a of part A of the figure, occurring at low deformations, due to orientation of the crystals of the γ form with chain axes nearly perpendicular to the stretching direction (vide infra). The azimuthal angle χ is defined in the image a of part A.

**Figure 2:**
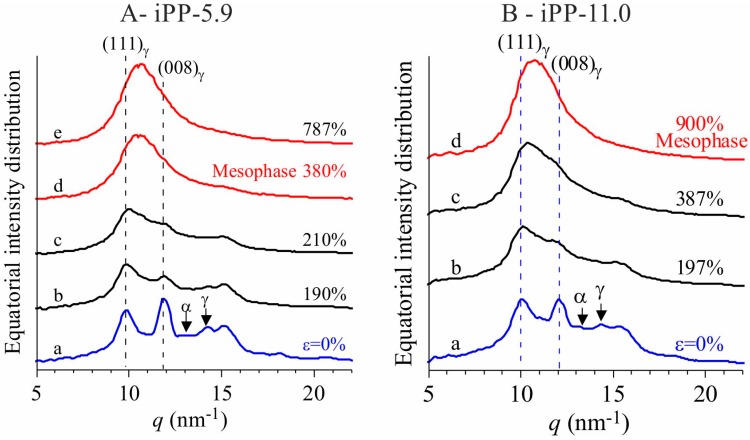
Equatorial profiles extracted from the bidimensional WAXS images of Figure 1 for the samples iPP-5.9 (**A**) and iPP-11.0 (**B**). The relevant reflections of α and γ forms are indicated.

The stress-strain curves and representative WAXS images collected in situ during stretching of compression-moulded specimens of the samples iPP-5.9 and iPP-11.0 are shown in Figure 1. The equatorial profiles extracted from the bidimensional WAXS images collected at relevant deformations are reported in Figure 2. The initial compression-moulded samples are crystallized from the melt in the γ form almost completely (patterns a of Figure 1) [19,20].

The WAXS images indicate that the γ form gradually transforms into the mesomorphic form of iPP by effect of deformation [16,17,18,19,20]. The transformation into mesophase starts at deformation ε_start_ ≈ 200% and is complete at ε_end_ ≈ 400–500% (Figure 1 and Figure 2). In all cases a highly oriented fibrillary morphology is obtained already at the deformations marking the end of transition [19,20].

**Figure 3:**
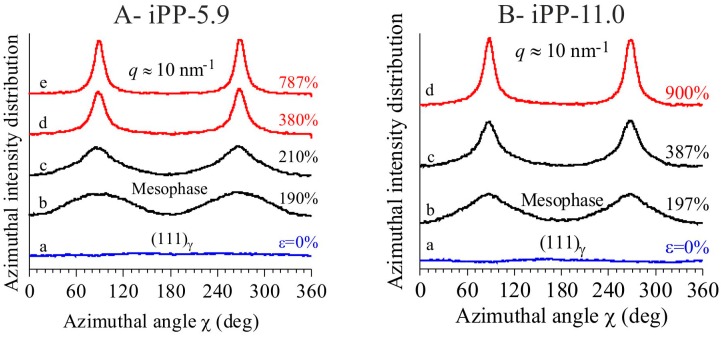
Azimuthal profiles of intensity centered in the *q* region around 10 nm^−1^, extracted from the bidimensional WAXS images of Figure 1 for the samples iPP-5.9 (**A**) and iPP-11.0 (**B**).

The textural transformations of the initially isotropically oriented morphology into a well-oriented fiber morphology is indicated by the change in distribution of intensity of the reflections along the azimuthal angle χ defined in the image a of Figure 1A. This is particularly evident for the changes in the azimuthal spreading of intensity at *q* ≈ 10 nm^−1^, corresponding to the reflection (111)_γ_ of γ form at low deformations (images a–c of Figure 1 and curves a of Figure 2), and to the tail of the mesomorphic halo centered at *q* ≈ 11 nm^−1^ at high deformations (images d–e of Figure 1A, d of Figure 1B, curves d–e of Figure 2A, and d of Figure 2B). In fact, as shown in Figure 3, the azimuthal intensity distribution at *q* ≈ 10 nm^−1^ is uniform for the undeformed samples (curves a of Figure 3) and becomes polarized on the equator with increasing the deformation (curves b–e of Figure 3A and b–d of Figure 3B).

**Figure 4:**
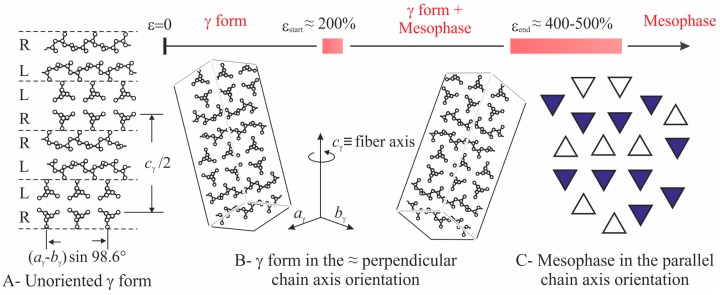
Structural model of γ form (**A**), scheme of the perpendicular chain axis orientation of γ crystals (**B**) and of mesomorphic domains of iPP (**C**), as they develop by effect of stretching, starting from unoriented specimens.

The intensity at *q* ≈ 12 nm^−1^ relative to the (008)_γ_ reflections of γ form (curves a of Figure 2), instead, at low deformations is polarized on a layer line off the equator (horizontal direction) and off the meridian (vertical direction), as indicated by the arrows in the images b, c of Figure 1, and only when the transformation into the mesophase is complete, that is, at deformations higher than ε_end_, the intensity at *q* ≈ 12 nm^−1^ becomes polarized on the equator [19,20]. This indicates that part of the crystals of the initial γ form tend to become oriented at low deformations with the chain axes nearly perpendicular to the stretching direction, as shown in Figure 4, instead than parallel as in the standard fiber morphology [16,38]. It is worth noting that for the perpendicular chain axis orientation of γ form, the polarization of the (111)_γ_ (at *q* ≈ 10 nm^−1^) reflection occurs on the equator [39].

## 4. Data Analysis and Discussion

The change of orientation of crystals by stretching is quantitatively analyzed using the azimuthal intensity at *q* ≈ 10 nm^−1^ of Figure 3. A numerical descriptor *O* that plays the role of an orientation order parameter is introduced from the calculation of the orientation function <*P*_2_> [40]. In general, for uniaxially symmetric materials (such as fibers) the orientation function for a direction normal (pole) to any given {*hkl*} family of planes <*P*_2_(cosχ)> with respect to a preferred direction (fiber axis) is defined as:

(1) $$\langle P_{2}\left(\cos\chi \right)\rangle =\frac{1}{2}\left(3\langle \cos^{2}\chi \rangle -1\right)$$

where <cosχ> is the average cosine of the angle χ that the poles make with the preferred direction and denotes the second order Legendre polynomial of argument <cosχ>. The values of <cosχ>, in turn, are calculated from the azimuthal intensity distribution of the *hkl* reflection as [40]:

(2) $$\langle \cos^{2}\chi \rangle =\frac{\int_{0}^{\pi /2}I(q)\cos^{2}\chi \sin\chi d\chi }{\int_{0}^{\pi /2}I(q)\sin\chi d\chi }$$

According to Equation (1), <*P*_2_> = 1 corresponds to an ideal case of perfect alignment of the poles in the preferential direction, <*P*_2_> = 0 corresponds to isotropic case and <*P*_2_> = −0.5 corresponds to an ideal case of perfect perpendicular orientation. 

In our case, the order parameter is calculated considering the degree of polarization of the intensity at *q* ≈ 10 nm^−1^ on the equator, with respect to the fiber axis. This would correspond to values of the orientation function <*P*_2_>_exp_ ranging from zero at zero deformation (completely isotropic case) to values less than zero with increasing the deformation. The parameter *O* is calculated by comparing the experimental values of <*P*_2_>_exp_ at *q* ≈ 10 nm^−1^ with that of an ideal model characterized by an extremely narrow equatorial polarization of intensity at this *q* (that is <*P*_2_>_id_ = −0.5) as:

(3) $$O=\frac{{\langle P_{2}\rangle }_{\exp}}{{\langle P_{2}\rangle }_{\mathrm{id}}}$$

According to Equation (3), the values of *O* are positive and range from zero (fully isotropic case) to 1 (fully oriented case).

**Figure 5:**
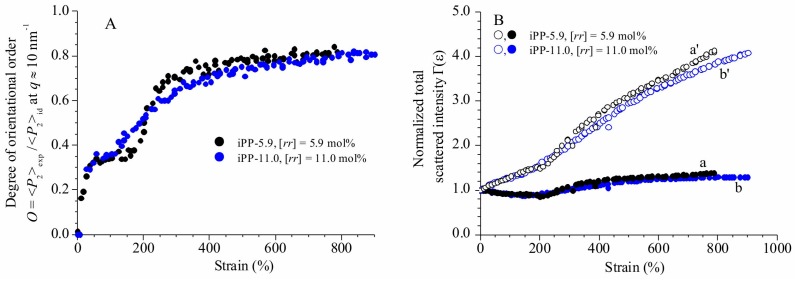
Orientation parameter *O* (**A**) and total WAXS scattered intensity (invariant) normalized for the integrated intensity of the unoriented sample Γ(ε) (**B**) as a function of strain, for the samples iPP-5.9 (○, ●) and iPP-11.0 (○, ●). The parameter *O* is calculated from the azimuthal intensity distribution at *q* ≈ 10 nm^−1^ in the bi-dimensional WAXS images of Figure 1, corresponding to the reflection (111)_γ_ of γ form at low deformations, to the tail of the mesomorphic halo centered at *q* ≈ 11 nm^−1^ at high deformations. It represents a measure of the average degree of orientation of the chain axes with respect to the fiber axis at high deformations (>ε_end_), and to the average degree of orientation of the residual crystals of γ form in the perpendicular chain axis orientation, and of the transformed mesomorphic domains in the parallel chain axis orientation at deformations lower than ε_end_. In (**B**), the curves a’ and b’ (open symbols) are obtained by multiplying the curves a and b (full symbols) by the factor *t*_0_/*t* = (*l*/*l*_0_)^1/2^ to account for the thickness contraction (see Experimental section).

Since the polarization intensity on the equator at *q* ≈ 10 nm^−1^ originates from the perpendicular chain axis orientation of the residual γ form crystals at low and moderate deformations and from the parallel chain axis orientation of the already transformed mesomorphic domains at moderate and high deformations, the value of the parameter *O* represents a measure of the average degree of orientation of the chain axes with respect to the fiber axis only at high deformations, that is, after complete transformation of the original crystals of γ form into the mesophase, whereas at lower deformation, the values of *O* represents a measure of the average degree of orientation of the residual crystals of γ form in the perpendicular chain axis orientation, and of the transformed mesomorphic domains in the parallel chain axis orientation. The so obtained values of the parameter *O* derived for the iPP samples are reported in Figure 5A. 

The evolution of orientational order *O* with deformation (Figure 5A) of the two samples follows a common trend involving different steps. For deformations ε lower than 100%, a steep increment of the orientational order occurs, due to orientation of the crystals in the γ form with chains axes perpendicular to stretching direction. In the deformation range between 100 and ≈150–200%, the values of *O* reach a quasi-plateau of 0.30–0.4. This indicates that at low deformations, changes in azimuthal intensity distribution of (111)_γ_ reflections of γ forms essentially probe small reorientation of the lamellar crystals along the pathway toward the fibrillary morphology, due to lamellar rotations [26,27,40]. The leading deformation mechanisms in the first plateau region, instead, correspond to interlamellar shear, that is slip of the crystalline lamellae parallel to each other, and/or interlamellar separation [26,27,28,41,42]. At this stage, no phase transitions occur and all movements are assisted by the shear deformation of the compliant interlamellar amorphous phase. Successively, starting from deformations close to the critical strain at which transformation into mesophase starts (ε_start_ ≈ 200%, Figure 1 and Figure 4), the orientational order *O* increases again up to reach a final plateau value of ≈0.8 at high deformations (Figure 5A). This indicates that after lamellar breaking, further deformation induces collective shear process, up to reach destruction of the preexisting crystals (mechanical melting) and successive recrystallization with formation of fibrils including mesomorphic aggregates [24,25].

A further quantitative descriptor of the changes at WAXS length scale is obtained using the ratio Γ(ε) = *Q*(ε)/*Q*(0) between the total WAXS intensity *Q*(ε) at deformation ε and the total scattered intensity *Q*(0) at ε = 0. For samples exhibiting uniaxial symmetry (fibers), the total scattered intensity in the WAXS region *Q*(ε) can be calculated from the WAXS intensity collected on a bidimensional detector (Figure 1) by integrating all over the sampled reciprocal space, that is between *q*_min_ = 3 nm^−1^ and *q*_max_ = 22 nm^−1^, using Equation (4):

(4) $$Q\left(\varepsilon \right)=\frac{1}{\pi }\int_{q_{\min}}^{q_{\max}}q^{2}\left(\int_{0}^{\pi }I_{\varepsilon }\left(q,\chi \right)\sin\chi d\chi \right)-Bk\left(q\right)dq$$

In Equation (4), it is implicitly assumed a uniform distribution of intensity along circles of radius *q* sinχ because of the cylindrical symmetry of the fibers. Moreover, the intensity, at any *q*, was subtracted for the background contribution (*Bk*(*q*)) estimated from the equation of the straight line subtending the radially averaged profiles extracted from the bi-dimensional diffraction images. The ratio of the total scattered intensity and the integrated intensity of the unoriented sample gives the normalized value of the total scattered intensity:

(5) $$\Gamma (\varepsilon )=\frac{Q(\varepsilon )}{Q(\varepsilon =0)}$$

Equation (4) corresponds to a reduced scattering invariant [43]. The values of the parameter Γ(ε) are reported in Figure 5B as a function of deformation before (curves a, b) and after (curves a’, b’) correction for the thickness contraction. 

Also in this case, we observe that the change of the parameter Γ(ε) with deformation follows a common trend for the two samples (Figure 5B). In particular, before correction for the thickness contraction, Γ(ε) decreases with increasing deformation up to ε ≈ 200%, then increases for further increment of deformation, up to reach a quasi-plateau at values close to 1.3–1.4 (curves a, b of Figure 5B). The decrease in Γ(ε) values of Figure 5B at deformations lower than 200% can be attributed to a decrease in the sample thickness. In fact, after correction for this effect only an increase of Γ(ε) with the deformation is observed (curves a’, b’ of Figure 5B). In particular, we use a correction factor equal to *t*_0_/*t* to account for the thickness contraction, by setting *t*_0_/*t* = (*l*/*l*_0_)^ν^ with ν = 0.5 (see Section 2). Therefore, curves a, b and a’, b’ of Figure 5B illustrate the change of the parameter Γ(ε) with increasing the deformation, in the limiting cases of no thickness contraction (Poisson’s ratio = 0, curves a, b) and in the rubbery limit of thickness contraction, corresponding to Poisson’s ratio ν = 0.5 (curves a’, b’). The invariant is expected to change with deformation according to a behavior in between these two limiting cases. 

After correction for the thickness contraction, the values of the WAXS reduced scattering invariant experience a remarkable increase with increasing the deformation, reaching values up to ≈4 times higher than those of the undeformed state. The remarkable increase of invariant with increasing deformation holds also after taking into account the absorption, since the correction of the scattered intensity for the transmission factor would decrease the values of the parameter Γ(ε) at most by a factor of 1.1. 

The increase of the total scattering intensity in the WAXS region by a factor 1.3–1.4 in the hypothesis of no thickness contraction, and a factor ≈4 in the hypothesis of thickness contraction according to the rubbery limit is unexpected. In fact, for a multi-phases system (in our case three phases, γ form, mesophase and amorphous component), the measured WAXS intensity *I*_ε_(*q*,χ) can be written as a linear combination of the scattering intensities from each phase:

(6) $$I_{\varepsilon }(q,\chi )=\sum_{i=1}^{3}x_{i}\left(\varepsilon \right)I_{\varepsilon i}(q,\chi )$$

where *I*_ε*i*_(*q*,χ) are the scattering intensities of the γ form (phase *i* = 1), mesophase (phase *i* = 2) and amorphous component (phase *i* = 3) and *x_i_*(ε) are the corresponding mass fractions. Therefore, also the total WAXS reduced invariant *Q*(ε) (Equation (4)) is a linear combination of the contribution from components *Q_i_*(ε).

(7) $$Q\left(\varepsilon \right)=\overset{3}{\underset{i=1}{\sum }}Q_{i}\left(\varepsilon \right)$$

Each term of the invariant *Q_i_*(ε), in turn, is proportional to the following quantities [44,45]:

(8) $$\;Q_{i}\left(\varepsilon \right)=\rho_{i\varepsilon }^{2}N_{i\varepsilon }V_{i\varepsilon }$$

where *ρ_i_* and *N_i_*_ε,_ are the electron density and the number of domains of phase *i* with average volume of coherence equal to *V_i_*_ε_,. The *N_i_*_ε_ domains contribute additively to the scattering, whereas the coherent (elastic) scattering occurs only within each domain. The density of γ form, mesophase and amorphous phase in isotactic polypropylene are 0.939, 0.91 and 0.854 g/cm^3^ [46] and the corresponding values of electron density are 323, 313 and 294 electrons/nm^3^ respectively. Based on previous analyses [16,17,18], the total degree of crystallinity, due to the γ form and mesophase contributions, apparently, does not greatly change during deformation (*x*_1_(ε) + *x*_2_(ε) ≈ 0.50, see Table 1). Therefore, in the hypothesis that also the total volume of the coherently scattering crystalline domains does not change by effect of deformation (*N*_1ε_ *V*_1ε,_ + *N*_2ε_ *V*_2ε,_ = cost.), the scattering invariant is expected to remain constant or to slightly decrease with increasing the deformation, since the γ form transforms into the mesophase with slightly lower electron density. 

**Figure 6:**
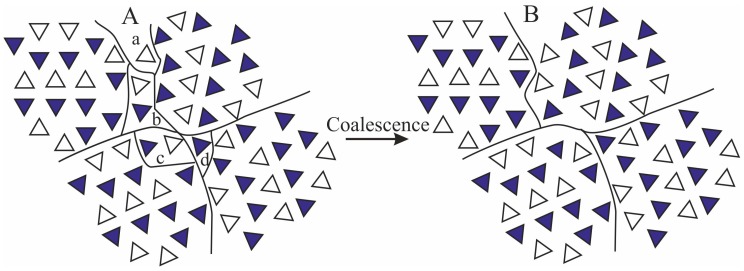
Model of coalescence of mesomorphic domains separated at the boundaries by isolated groups of 1–2 chains (sub-domains a–d) in a completely different arrangement (**A**) with formation of larger mesomorphic domains (**B**). By effect of stretching, the sub-domains a–d coalesce with close-neighbors mesomorphic domains.

The tendency of the scattering invariant to increase at deformations lower than ε_start_ marking the beginning of transformation of the initial γ form into mesophase, may be due to local “densification” of the amorphous intralamellar phase by effect of stretching, since the intralamellar amorphous phase experiences compressive forces in the direction parallel or perpendicular to the layers, depending on the orientation, perpendicular or parallel to the stretching direction, respectively, of the lamellar stacks in which the amorphous layers are embedded [2,20]. At deformations higher than ε_start_, marking also the beginning of lamellar destruction, we hypothesize that the increase of invariant may be due to a slight increase of the size of coherent domains located in the mesomorphic aggregates, leading to a remarkable increase of their average volumes *V*_2ε_, even though the total number of these domains *N*_2ε_ does not necessarily change. This increase may involve the incorporation of isolated chains, or groups of 2–3 chains located at the boundaries of adjacent domains, into close neighboring larger domains through small movements and/or rotations facilitated by deformation, as shown in the model of Figure 6.

Moreover, considering that the mesophase originates from the breaking of crystals of γ form through a process of mechanical melting and re-crystallization, and that the lamellar crystals of γ form are not characterized by chain folding because of the non-parallel arrangement of the chains (Figure 4A) [47], the size of the coherent domains in a mesomorphic aggregate may easily increase by effect of stretching also in the direction parallel to the chain axes, through incorporation of monomeric units located at the boundaries with the amorphous phase. We calculate that, on average, an increase of the coherent length by a factor of 1.1–1.6 along the three orthogonal directions *x*, *y* and *z* of a domain, leads to an increase of volume by a factor 1.1^3^–1.6^3^ ≈ 1.3–4. As shown in the model of Figure 6, the increase of the coherent length of the mesomorphic domains by incorporation (coalescence) of the isolated sub-domains a-d within the mesomorphic domains, does not necessarily lead to a decrease of the number of domains *N*_2ε_. As a consequence, the total scattering intensity increases by effect of deformation, because the product (*N*_2ε_*V*_2ε_) increases due to coalescence phenomena of the kind depicted in Figure 6. 

**Figure 7:**
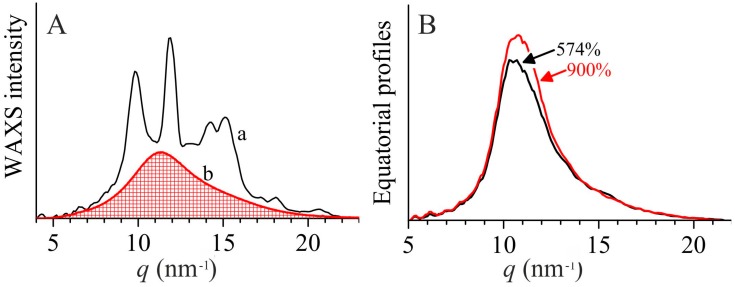
(**A**) Radially averaged WAXS profile of the undeformed sample iPP-5.9, extracted from the bidimensional WAXS image a of Figure 1A (a) and underlying amorphous contribution, approximated by the diffraction profile of an atactic polypropylene (b). (**B**) Equatorial profiles extracted from the bidimensional WAXS images of Figure 1B, of the sample iPP-11.0 stretched at 574 and 900% deformations. The diffraction curves are subtracted for the background contribution, approximated as a straight line subtending the whole profiles.

On the other hand, the fact that the crystallinity index measured from the radially averaged WAXS profiles as the ratio between the area of the crystalline phase (*A*_c_) and the total area of the diffraction profile (*A*_tot_) does not apparently change (see Figure 7A), is due to the fact that *A*_c_ is often overestimated in this procedure, as it is obtained by the difference of *A*_tot_ and the diffraction area from the amorphous component (approximated in Figure 7A by the diffraction profile of an atactic polypropylene, curve b). In other terms, the crystallinity calculated from the radially averaged WAXS curves according to the procedure of Figure 7A, includes not only the Bragg contribution, but also diffuse scattering located in the diffraction region between the amorphous halo and the Bragg peaks (Figure 7A). Instead, in the experimental value of the scattering invariant, the diffuse scattering terms makes a contribution, which is at least one order of magnitude lower than the Bragg component. Diffuse scattering arises from the presence of disorder and its contribution to the scattering intensity is proportional to the fluctuations of the structure factor *f* (per monomeric unit), given by the term <*f* ^2^> − <*f*>^2^, which represent the difference between the average square (<*f* ^2^>) and the square of average (<*f*>^2^) of *f* [48]. Therefore, the higher the local structure factor deviates from the average value <*f*> because of the presence of disorder, the higher the fluctuations, and the higher the diffuse scattering. By effect of coalescence of the small sub-domains into the large domains, the diffuse scattering decreases and the interference term (Bragg-like contribution) increases. The WAXS invariant increases, because the diffuse scattering term is always lower than the Bragg term.

In order to probe the effective increase of the size of the mesomorphic domains by effect of stretching, we compare in Figure 7B the equatorial profiles of the sample iPP-11.0 stretched at 574 and 900% deformations. The curves are not corrected for the thickness contraction, but only for the transmission factor. Yet, it is evident that at 900% deformation the equatorial peak of the mesophase becomes narrower than that at 574% deformation, confirming the increase of the lateral size of the mesomorphic domains. It is worth noting that the maximum is shifted toward slightly higher *q* values at 900% deformation, indicating that the average correlation distance between the chain axes within the mesomorphic domains decreases, producing increase of electron density. Therefore, the increase of the scattering invariant is not only due to coarsening, but also to increase of electron density (densification).

## 5. Conclusions

The structural transformations occurring by effect of stretching in stereo-irregular isotactic polypropylene samples are studied in situ during deformation by collecting WAXS patterns. The metallocene-made iPP samples selected in this study show high ductility, allowing achieving high deformations before breaking. 

WAXS analysis reveals that the crystals of γ form tend to assume a nearly perpendicular chain axis orientation instead than parallel to the stretching direction at low deformations. At ≈200% deformation (ε_start_) the γ form starts transforming into the mesomorphic form of iPP, and at 400–500% deformation (ε_end_), the transformation is almost complete. The stress induced phase transition of the γ form into the mesophase occurs after the beginning of lamellar fragmentation, concomitant with destruction of the crystalline blocks (mechanical melting) and re-crystallization in fibrillary entities containing mesomorphic aggregates. At the end of transformation into mesophase, a fibrillary morphology develops with a high degree of orientational order of the chain axes parallel to the stretching direction. We show that the chains extracted from the original crystals re-crystallize forming aggregates of mesomorphic domains responsible for the coherent elastic scattering (Bragg-like contribution) which include at the boundaries isolated chains or groups of 2–3 chains, which contribute to diffuse scattering. The coalescence process of these chains with the larger mesomorphic domains, increases the volume of coherence of the mesomorphic domains. Since the number of coherent domains does not decrease, the WAXS scattering invariant increases by effect of coalescence during deformation, even if the sampled *q* region is narrow. Coalescence starts concomitant with the process of lamellar destruction, and beginning of transformation of the initial γ form into the mesophase and, after complete transformation into the mesomorphic form, continues facilitated by the mechanical stress field.

## Figures and Tables

**Table 1:** Molecular mass (*M*_v_), melting temperature (*T*_m_) and content of *rr* triads of iPP samples prepared by metallocene catalysts as described in ref. [22] ^a^.

Sample	*M*_v_ (kg/mol) ^b^	*T*_m_ (°C) ^c^	[*rr*] (mol %) ^d^	x_c_^f^ (%)	*f*_γ_^f^ (%)
iPP-5.9	211	114	5.92	55	96
iPP-11.0	123	84	11.01	42	100

^a^ The polydispersity index of molecular masses is close to 2. ^b^ From the intrinsic viscosities [15,22]. ^c^ The melting temperatures were obtained with a differential scanning calorimeter Perkin Elmer DSC-7 performing scans in a flowing N_2_ atmosphere and heating rate of 10 °C/min [22]. ^d^ Determined from solution ^13^C NMR analysis [22]. ^f^ Crystallinity index x_c_ and relative amount of γ form with respect to the α form *f*_γ_ evaluated from X-ray diffraction profiles of compression-moulded samples [16,17,18,19,20].
